# Traditional Chinese medicine Jianpi therapy in exercise-induced fatigue: A protocol for systematic review and meta-analysis

**DOI:** 10.1097/MD.0000000000028594

**Published:** 2022-01-14

**Authors:** Xue Geng, Xiujuan Guo, Baoquan Liu, Peiying Yu, Jiazhou Li, Huashan Pan

**Affiliations:** a School of Physical Education and Health of Guangzhou University of Chinese Medicine, China; b Guangzhou University of Chinese Medicine, China; c Guangdong Chaozhou Health Vocational College, China.

**Keywords:** exercise-induced fatigue, Jianpi therapy, network meta-analysis, protocol, systematic review, traditional Chinese medicine

## Abstract

**Background:**

Exercise-induced fatigue (EIF) is a common occurrence in sports competition and training. It may cause trouble to athletes’ motor skill execution and cognition. Although traditional Chinese medicine Jianpi therapy has been commonly used for EIF management, relevant evidence on the effectiveness and safety of Jianpi therapy is still unclear.

**Methods:**

Databases including PubMed, Embase, Web of Science, the Cochrane Library, SinoMed, China Science and Technology Journal Database (VIP), China National Knowledge Infrastructure (CNKI), and Wanfang will be searched for relevant randomized controlled trials from databases from 2000 to 2021. Randomized controlled trials related to traditional Chinese medicine Jianpi therapy in the treatment and management of EIF will be included. Systematic review and meta-analysis of the data will be performed in RevMan 5.3 according to the Preferred Reporting Items of Systematic Reviews and Meta-Analysis (PRISMA) guidelines. Two authors independently performed the literature searching, data extraction, and quality evaluation. Risk of bias was assessed using the Cochrane Risk of Bias Tool for randomized clinical trials.

**Results:**

This systematic review and meta-analysis will summarize the latest evidence for traditional Chinese medicine Jianpi therapy in EIF. The results will be submitted to a peer-reviewed journal once completed.

**Conclusion:**

The conclusion of our research will provide evidence to support traditional Chinese medicine Jianpi therapy as an effective intervention for patients with EIF.

OSF Registration DOI: 10.17605/OSF.IO/NRKX4.

## 1 Introduction

Exercise-induced fatigue (EIF) is a reduction in maximal voluntary muscle force that results from intense and prolonged exercise,^[1]^ which becomes a ubiquitous phenomenon in sports competition and physical training.^[2]^ EIF develops after a long time or intense physical exercise resulting from a reduction of skeletal muscle contractile function, during highly intense exercise skeletal muscle fibers of athletes have to rely on anaerobic metabolism leading to acute sports fatigue.^[3]^ As a symptom of exhaustion, the feelings of fatigue not only occur in chronic and acute disease conditions but also during sustained strenuous exercise. The underlying mechanisms of EIF in diseases seem to be closely related to the neuroinflammatory pathways.^[4]^ Studies had shown that impaired control of eye movements of athletes following fatiguing exercise. Prolonged use of the skeletal motor system has an adverse effect on the function of the oculomotor system, suggesting a potential role of central fatigue.^[5]^ Even worse, EIF may damage athletes’ motor skill execution and cognition ability, the underlying cellular mechanism of movement control and motor learning is related to corticostriatal synaptic plasticity.^[6]^ EIF resulting from intense and prolonged exercise without sufficient recovery will not only influence the motor skill execution but also damage the synaptic plasticity and cognitive function, such as attention span, information processing and decision making.^[7]^

In recent years, studies have shown that traditional Chinese medicine Jianpi therapy can improve the clinical efficacy in the treatment of EIF.^[8,9]^ However, there still exists some limitations like single reports, scattered research, small sample size. Systematic reviews and meta-analyses are fundamental tools for the generation of reliable summaries of health care information for clinicians, decision makers, and patients.^[10]^ However, to our knowledge, no systematic review and meta-analysis have reported the effect of traditional Chinese medicine Jianpi therapy in EIF. Although traditional Chinese medicine Jianpi therapy has been commonly used for EIF management, relevant evidence on the effectiveness and safety of Jianpi therapy is still unclear. The current study aims to evaluate the efficacy and safety of traditional Chinese medicine Jianpi therapy in EIF.

## 2 Materials and methods

### 2.1 Study registration

We had registered this systematic review on the OSF registration platform and got the Registration DOI: 10.17605/OSF.IO/NRKX4. This systematic review and meta-analysis protocol followed the guidelines including Preferred Reporting Items for Systematic Reviews and Meta-Analyses Protocols (PRISMA-P).^[11]^ We used the Population-Intervention-Comparators-Outcomes-Study design framework (PICOS) to guide the eligibility criteria. Ethical approval was not required.

### 2.2 Eligibility criteria

(1) Participants: patients who were clearly diagnosed with EIF, and gender and age were not limited.
(2) Study type: Randomized controlled trials (RCTs) of traditional Chinese medicine Jianpi therapy intervention in EIF patients will be concluded in our systematic review and meta-analysis. Language limited to Chinese and English.
(3) Interventions: The observation groups received pure traditional Chinese medicine Jianpi therapy in EIF, or combined with conventional treatment. Controlled interventions included any other positive interventions (no Chinese medicine Jianpi therapy treatment), rest relief, waiting, placebo, etc.
(4) Outcomes: Primary outcome measures included the rating of perceived exertion as subjective scale, 800-m race performance and Harvard Step Index as objective assessment. Secondary outcome measures were defined as hemoglobin and blood lactic acid as biochemical outcomes and immediate postexercise heart rate as the physiological outcome. It is required to report at least one of the main outcome indicators listed in the included studies.
(5) Exclusion criteria: The detailed data or important materials are missing; the data analysis cannot be performed. We will select the literature with the most complete data for those repeated publications. The type of publications were comments, experience presentations, or case reports will be excluded.

### 2.3 Searching strategy

Strategy for clinical studies recommended by Cochrane Handbook was applied. Besides, PICOS model (P = patients/population, I = target intervention, C = controlled intervention, O = outcome, S = study) was considered. Databases including PubMed, Embase, Web of Science, the Cochrane Library, SinoMed, China Science and Technology Journal Database (VIP), China National Knowledge Infrastructure (CNKI), and Wanfang will be searched for relevant RCTs from databases from 2000 to 2021. RCTs related to traditional Chinese medicine Jianpi therapy in the treatment and management of EIF will be included. We will use the following combined text: “traditional Chinese medicine”, “Jianpi”, “Bupi”, “Yipi”, “exercise-induced fatigue”, “sports fatigue”, “fatigue”, “randomized controlled trials”, “RCT”.

### 2.4 Data extraction

Studies will be searched by 2 researchers independently. Disagreement in literature inclusion between them will either be solved through discussion or passed to the third researchers to make a decision. Collection items will cover the following items: article title, first author, year of publication, diagnosis criteria, sample size, age, gender, target intervention, controlled intervention, course of treatment, follow-up, outcomes, bias assessment, introduction on informed consent, statistical results, and adverse events.

### 2.5 Quality assessment

Quality of included RCTs will be assessed based on Cochrane Collaboration assessment tool for risk of bias,^[12]^ with items including: random sequence generation, allocation concealment, blinding of participants and personnel, blinding of outcome assessment, incomplete outcome data, selective reporting and other sources of bias. The judgment of each item is divided into 3 levels: low risk of bias, high risk of bias, and unclear risk of bias. The quality assessment will be performed independently by 2 researchers using Review Manager (version 5.3, Nordic Cochrane Centre, Cochrane Collaboration) based on the criteria of the Cochrane Risk of Bias Tool (version 5.1.0). We will assess the evidence of outcomes through the “grading of recommendations assessment, development, and evaluation (GRADE)” system. Including 5 aspects (bias, indirection, inconsistency, imprecision, and risk of publication bias). The GRADE profiler software (version 3.2, Evidence Prime) will be performed for analysis.

### 2.6 Statistical analysis

We will use Review Manager (version 5.3) to perform this systematic review and meta-analysis. Categorical variable and continuous variable outcomes are respectively used relative risk, mean difference to represent the effect index, and calculate the 95% confidence interval. *P* values and *I*^2^ values were both set statistical difference levels for heterogeneity test among studies.^[13]^ If *I*^2^ ≤ 50% and *P* ≥ .05, meta-analysis will be used to combine the effect size, fixed effect model was applied for mathematical synthesis; if *I*^2^ > 50%, *P* < .05, the meta-regression, subgroup analysis, and sensitivity analysis will be used to explore the source of heterogeneity, while random effect model will be used.

## 3 Discussion

With the continuous development of sports education around the world, the expectations of competitive sports are also increasing. In order to improve athletes’ control ability and their competitive level, it is necessary to relieve fatigue after exercise. EIF has received more and more attention from society and the country. The occurrence of EIF will directly affect the improvement of athletes’ sports ability, thereby affecting their sports performance. To quickly and effectively alleviate EIF is a key issue that needs to be considered in the development of sports, and it is also the focus of sports research in recent years. Therefore, it is of great significance to choose reasonable and effective methods and drugs to relieve EIF.

Traditional Chinese medicine Jianpi herb “Astragalus membranaceus”, a popular “Qi-tonifying” herb with a long history of use as a traditional Chinese medicine with multiple biological functions, improves exercise performance and ameliorates EIF, reduces exercise-induced accumulation of the byproducts blood lactate and ammonia with acute exercise challenge.^[14]^ Ginseng, a famous traditional Chinese medicine Jianpi therapy, ameliorates EIF potentially by regulating the gut microbiota, mechanistically, the saccharides and ginsenosides in Ginseng play an important role in the treatment of EIF, they serve as energy substrates for specific intestinal bacteria, thereby usefully regulating the gut microbiota, most significantly, Ginseng triggers several unique and key molecular and cellular signaling pathways to perform the beneficial reaction on EIF.^[15]^ Research showed that Rhodiola Crenulata relieves exhaustive EIF by inhibiting mitophagy in skeletal muscle, mechanistically, it may be related to the improvement of antioxidant activity, enhancement of energy production and suppression of mitophagy by inhibiting the PINK1/Parkin signaling pathway.^[16]^

The systematic review and meta-analysis protocol aims to evaluate the efficacy and safety of traditional Chinese medicine Jianpi therapy in EIF. The design of our research conforms to the guideline of Preferred Reporting Items for Systematic Review and Meta-Analysis Protocols (PRISMA-P). The results of this systematic review and meta-analysis will be submitted to a peer-reviewed journal.

## Author contributions

**Conceptualization:** Huashan Pan, Baoquan Liu.

**Data curation:** Xue Geng, Xiujuan Guo, Baoquan Liu.

**Formal analysis:** Xue Geng, Jiazhou Li.

**Funding acquisition:** Huashan Pan.

**Investigation:** Xue Geng, Xiujuan Guo, Peiying Yu, Huashan Pan.

**Methodology:** Xue Geng, Xiujuan Guo, Peiying Yu, Jiazhou Li.

**Project administration:** Huashan Pan.

**Software:** Xue Geng, Xiujuan Guo, Jiazhou Li.

**Supervision:** Huashan Pan.

**Validation:** Peiying Yu, Jiazhou Li.

**Writing – original draft:** Xue Geng, Xiujuan Guo.

**Writing – review & editing:** Huashan Pan.
